# Notes and News

**Published:** 1900-01-27

**Authors:** 


### Notes and News.

### HOSPITAL MEETINGS.

German Hospital, Dalston.—Annual Court at two p.m., on Friday, January 26th, at Winchester House, E C.

Italian Hospital.—Annual Meeting at half-past four, on Saturday, January 27th, at the Temporary Hospital, 2, Orange Street, W.C.

London Throat Hospital.—Annual Meeting at five p m., on Tuesday, January 30th, at the Hospital, 204, Great Portland Street.

General Lying-in Hospital, York Road, Lambeth.—Annual General Meeting at half-past four p.m., on Wednesday, January 31st, at the Hospital.

Another transport, the "Canada," has arrived at Southampton, bringing about 200 sick and wounded for Netley. We learn that the cases, for the most part, are very serious.

The recent additions and improvements at the West London Hospital were opened last week for inspection. A sum of £63,000 is stated to be required to bring up the extensions to the required accommodation for 250 beds and cots.

The Princess Christian visited the Warneford Hospital on the 17th instant to open the new Victoria wing, when she named the upper ward in the building the "Helena" ward. The new wing is the Jubilee memorial for Leamington and the district.

The Hygienic Exhibition at Naples, to be opened by the King of Italy in April, should attract many English people interested in sanitary science. The exhibits promise to be of considerable interest, and conferences will be held on subjects of the day, such as tuberculism, which is claiming a great deal of attention in Italy.

The proceedings at hospital meetings are showing more and more that an active interest is taken in the practical details of institutional management. At Hartlepool recently, for instance, the Governors observed that the price of certain provisions appeared to be high. A satisfactory explanation was forthcoming, and no doubt this would frequently be the case where similar inquiries are made. But the effect in any case is a good one, and careful expenditure is encouraged. None should undertake the office of Governor of an institution without studying the economics of management and the commissariat. There are plenty of useful publications to be had dealing with institutional matters.

A cottage hospital has been opened at Urmston in commemoration of the Diamond Jubilee. Two wards, containing three beds and two cots, are situated on the ground floor, and connected by a cross-ventilated passage with each is a sanitary block. The cost has been about £1,200.

It must be good news to those who are obliged to reside in Dublin that an inquiry as to the acknowledged unhealthiness of the city is to be made. The committee to be formed for the purpose will be drawn from officials of Irish medical and sanitary corporations, who will be assisted by an inspector of the English Local Government Board.

Sir Michael Foster has consented to become a candidate for the seat in Parliament vacated by Sir John Lubbock, who has been elevated to the Peerage. Sir Michael Foster is chairman of the committee of the University of London, and Professor of Physiology at Cambridge. He is therefore a very suitable candidate to represent the University of London.

The highest praise of the conduct of the members of the Royal Army Medical Corps reaches us. Not only is their indefatigable diligence and care for the comfort of the wounded commented upon, but the general exhibition of bravery under fire has provoked much comment. Many of the wounded have been assisted under hot fire, and ambulances have been frequently fired upon by the enemy, without hindering the steady work of the surgeons.

Mr. Peckover, Lord-Lieutenant of Cambridgeshire, has made a New Year's offering of £1,500 to the Eastern Counties Asylum for Idiots, to defray the cost of furnishing and structural additions to the Peckover Schools at the asylum. Mr. Peckover has devoted £6,000 to the new schools and workshops. Great interest is being evinced in the Yeomanry Hospital, and many firms have made most useful offerings in kind. The bedstead makers of Birmingham have given all the beds, and a large proportion of the blankets and linen have been given, too. Much is still needed, however, and a list can be obtained from the Countess of Essex, 20, Curzon Street, S.W.

Electric lighting in hospital work was hailed as an unmitigated blessing at the outset. The recent experiences at the Middlesex Hospital show that it can add greatly to the difficulties of the surgeon. The Metropolitan Company's service of electric light has been most irregular and inadequate during the past year, and not only have business and private houses suffered, but we learn that during operations the light has been suddenly cut off at the Middlesex Hospital. The sudden failure of light is in most circumstances highly inconvenient, but at a hospital the results may be most serious. In old buildings the gas has been laid on, and it is much wiser not to allow it to be cut off entirely anywhere, as the expense of utilising a small amount of gas and the electric light more universally is no greater than by lighting the whole of a building with electric light, which fails occasionally under the best of company management. Certainly in a hospial an operating theatre, at any rate, should have an alternate light available.

